# Peace, Rude Boreas

**Published:** 1868-08

**Authors:** 


### "PEACE, RUDE BOREAS."

The communication in the present number of the REGISTER bearing this title, is a pretty lengthy and severe criticism upon a criticism, in the May number of this journal, page 191.

The apparent personalities in both of these articles would have been sufficient to have excluded them, but from the fact, that they are interesting, and bring out and present in a forcible manner many points upon a subject that is now receiving much attention by the profession.

Controversy oftentimes brings out truth in such a way as to be much easier apprehended and applied, than if stated in a cold, didactic style. We would suggest to the writers, that if they have occasion to try swords again, that they take it rather more moderately. Remember it is hot weather now, and there is danger in violent exercise. A hunter once went out for game—he found it, and so great was his excitement, haste and determination to kill it, that, in addition to the ball, he shot his ramrod. The ball would have done just as well. T.

